# The Importance of Probe Location for the Interpretation of Cerebral Microdialysis Data in Subarachnoid Hemorrhage Patients

**DOI:** 10.1007/s12028-019-00713-8

**Published:** 2019-04-29

**Authors:** Mario Kofler, Maxime Gaasch, Verena Rass, Alois J. Schiefecker, Bogdan Ianosi, Anna Lindner, Ronny Beer, John F. Stover, Paul Rhomberg, Bettina Pfausler, Claudius Thomé, Erich Schmutzhard, Raimund Helbok

**Affiliations:** 1 Neurological Intensive Care Unit, Department of Neurology, Medical University of Innsbruck, Anichstrasse 35, 6020 Innsbruck, Austria; 2 Medical Informatics, UMIT – University for Health Sciences, Hall, Austria; 3 Fresenius Kabi, Else-Kröner-Straße 1, 61352 Bad Homburg vor der Höhe, Germany; 4 Department of Neuroradiology, Medical University of Innsbruck, Innsbruck, Austria; 5 Department of Neurosurgery, Medical University of Innsbruck, Innsbruck, Austria

**Keywords:** Cerebral microdialysis, Cerebral metabolism, Subarachnoid hemorrhage, Computed tomography, Secondary brain injury

## Abstract

**Background:**

There is no uniform definition for cerebral microdialysis (CMD) probe location with respect to focal brain lesions, and the impact of CMD-probe location on measured molecule concentrations is unclear.

**Methods:**

We retrospectively analyzed data of 51 consecutive subarachnoid hemorrhage patients with CMD-monitoring between 2010 and 2016 included in a prospective observational cohort study. Microdialysis probe location was assessed on all brain computed tomography (CT) scans performed during CMD-monitoring and defined as *perilesional* in the presence of a focal hypodense or hyperdense lesion within a 1-cm radius of the gold tip of the CMD-probe, or otherwise as *normal*-*appearing brain tissue*.

**Results:**

Probe location was detected in *normal*-*appearing brain tissue* on 53/143 (37%) and in *perilesional* location on 90/143 (63%) CT scans. In the perilesional area, CMD-glucose levels were lower (*p* = 0.003), whereas CMD-lactate (*p* = 0.002), CMD-lactate-to-pyruvate-ratio (LPR; *p* < 0.001), CMD-glutamate (*p* = 0.002), and CMD-glycerol levels (*p* < 0.001) were higher. Neuroglucopenia (CMD-glucose < 0.7 mmol/l, *p* = 0.002), metabolic distress (*p* = 0.002), and mitochondrial dysfunction (*p* = 0.005) were more common in perilesional compared to normal-appearing brain tissue. Development of new lesions in the proximity of the CMD-probe (*n* = 13) was associated with a decrease in CMD-glucose levels, evidence of neuroglucopenia, metabolic distress, as well as increasing CMD-glutamate and CMD-glycerol levels. Neuroglucopenia was associated with poor outcome independent of probe location, whereas elevated CMD-lactate, CMD-LPR, CMD-glutamate, and CMD-glycerol levels were only predictive of poor outcome in normal-appearing brain tissue.

**Conclusions:**

Focal brain lesions significantly impact on concentrations of brain metabolites assessed by CMD. With the exception of CMD-glucose, the prognostic value of CMD-derived parameters seems to be higher when assessed in normal-appearing brain tissue. CMD was sensitive to detect the development of new focal lesions in vicinity to the neuromonitoring probe. Probe location should be described in the research reporting brain metabolic changes measured by CMD and integrated in statistical models.

**Electronic supplementary material:**

The online version of this article (10.1007/s12028-019-00713-8) contains supplementary material, which is available to authorized users.

## Introduction

Cerebral microdialysis (CMD) is a powerful monitoring tool providing insight into cerebral metabolic changes in patients suffering from acute brain injury [1]. In poor-grade subarachnoid hemorrhage (SAH) patients, research has focused on the detection of delayed cerebral ischemia (DCI) and the prediction of functional outcome [2]. Recent studies also demonstrated its utility in elucidating mechanisms of early brain injury [3], monitoring the effects of interventions on cerebral metabolism, and guiding systemic glucose management [2, 4, 5].

Current guidelines recommend CMD-catheter placement in the frontal watershed of the hemisphere ipsilateral to the aneurysm or in the vascular territory deemed at the greatest risk of developing secondary brain injury [1]. Catheter location should be reported in all research manuscripts reporting CMD-data, as CMD is a local monitoring method and concentrations of brain metabolites depend on focal brain pathology surrounding the probe. So far, no uniform recommendation of how to classify catheter location exists, resulting in a large heterogeneity of definitions in the literature [2]. Marked differences in CMD-derived metabolites in normal-appearing and (peri-)lesional brain tissue have been reported in traumatic brain injury (TBI) patients [6–9]. Accordingly, changes detected in the perilesional area may need to be interpreted differently than in normal-appearing brain tissue and even imply separate specific treatment consequences. Furthermore, it is unclear how CMD-derived biomarkers are associated with outcome depending on probe location [10].

Focal brain lesions after SAH include hyperdense and hypodense areas on brain computed tomography (CT), reflecting parenchymal hemorrhage, focal edema, and ischemia. Acute focal neurological deficits were associated with differences in cerebral metabolism, but the authors did not report spatial relation of CMD-probes to focal brain lesions [11]. The importance of CMD-probe location in the detection of silent cerebral infarcts has also been reported [12]. Changes in brain metabolism were detected hours preceding the detection of the infarct by head CT scans when probes were in the vicinity of the ischemic tissue; to the contrary, CMD was unable to detect ischemia when probe location was remote from the lesion (contralateral hemisphere or > 4 cm) [12].

In this study, we aimed to investigate the impact of catheter location on brain interstitial metabolite concentrations using a systematic approach and to study temporal dynamics in patients developing focal pathologies during monitoring time. We hypothesized that patterns of ischemia and metabolic derangement would be more common in the vicinity of focal brain lesions and that the association between cerebral metabolism and functional outcome of patients would depend on catheter location.

## Methods

### Patient Selection

This is a retrospective analysis of prospectively recorded observational data of 51/64 consecutive SAH-patients fulfilling the inclusion/exclusion criteria admitted to the neurological intensive care unit at the Medical University of Innsbruck, Austria, requiring multimodal neuromonitoring between 2010 and 2016. According to our institutional policy, invasive neuromonitoring is only performed in mechanically ventilated patients (poor admission grade or secondary deterioration). The study was approved by the local ethics committee, has been performed in accordance with the ethical standards as laid down in the 1964 Declaration of Helsinki. Informed consent was obtained from all patients according to federal regulations. Inclusion criteria were (1) admission with non-traumatic SAH, (2) ≥ 18 years of age, (3) neuromonitoring including CMD and (4) at least one brain CT scan during the time of CMD-monitoring. We excluded patients in whom a CMD-catheter was inserted but was dysfunctional (i.e. did not yield samples *n* = 7), patients lacking head CT scans during CMD-monitoring measurements would rather represent concentrations in blood, not in brain tissue, *n* = 3), leaving 51 patients eligible for analysis.

### Grading and Patient Care

Disease severity was graded using the Hunt and Hess scale and the Acute Physiology and Chronic Health Evaluation II score [13, 14]. Head CTs were performed on admission, after aneurysm treatment and when clinically needed and graded by an independent neuroradiologist (PR) using the modified Fisher score, SAH-sum score, and intraventricular hemorrhage sum score and were assessed for the presence of global cerebral edema (GCE) [15–17]. Clinical care was based on current international guidelines [18, 19], with the exception of continuous intravenous application of nimodipine. Ruptured aneurysms were treated by surgical clipping or endovascular coiling. Intravenous fluids (crystalloids and colloids), vasopressors (noradrenalin and phenylephrine), and dobutamine were used for hemodynamic stabilization. All patients were comatose during the period of invasive neuromonitoring and routinely received continuous intravenous midazolam and sufentanil. Patients were followed with transcranial color-coded duplex sonography. DCI was defined as new infarct on CT or magnetic resonance imaging (MRI), not attributable to other causes [20]. Functional outcome was assessed 3 months after SAH using the modified Rankin scale (mRS). In view of the high proportion of poor functional outcome in poor-grade SAH-patients, a mRS-score of ≤ 3 was defined as good functional outcome.

### Data Collection and Neuromonitoring

Patient characteristics, interventions, complications, and outcome were prospectively recorded in our institutional SAH-database. Continuous parameters were recorded in our patient data management system (Centricity™ Critical Care 8.1 SP7; GE Healthcare Information Technologies, Dornstadt, Germany) and meaned over the CMD-sampling time (1 h). Based on clinical and imaging criteria, patients received multimodal neuromonitoring including CMD. The CMD-probe (71 High Cut-Off Brain Microdialysis Catheter, membrane length 1 cm, pore size 100 kDa) was tunneled and placed in the white matter of the frontal watershed ipsilateral to the aneurysm or the vascular territory exhibiting the maximal pathology. Not more than 1 CMD-catheter was inserted per patient. Isotonic perfusion fluid [Perfusion Fluid CNS (central nervous system), Mdialysis™] was used at a flow rate of 0.3 μl/min. CMD samples were immediately analyzed with CMA 600 or Iscus^flex^ (all CMD-equipment by M Dialysis AB, Stockholm, Sweden) for CMD-glucose, CMD-pyruvate, CMD-lactate, CMD-glutamate, and CMD-glycerol concentrations. The first sample was drawn at least 1 h after probe insertion and discarded to avoid artefacts. Metabolic distress was defined as a lactate-to-pyruvate ratio (LPR) > 40, mitochondrial dysfunction as LPR > 30 together with CMD-pyruvate > 70 µmol/l, and neuroglucopenia as CMD-glucose < 0.7 mmol/l.

## Probe Location

**Fig. 1:**
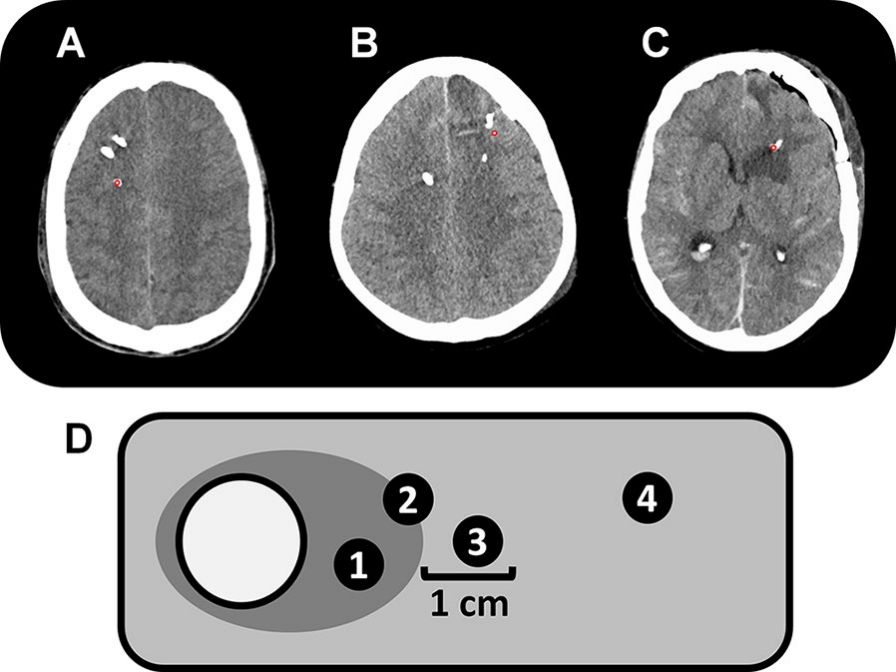
Examples for probe location in normal-appearing brain tissue (**a**), perilesional (**b**), and intralesional (**c**) probe location on axial computed tomography scans of the brain; panel **d** is a schematic diagram describing probe location classification. The white circle represents a parenchymal hemorrhage with perifocal edema (dark gray area), surrounded by normal-appearing brain tissue (light gray area). Tips of microdialysis probes completely surrounded (location 1) or in direct contact with (location 2) a hypodense area were classified as intralesional. Perilesional location was defined as lesion within 1 cm of the catheter tip (location 3). Location 4 represents normal-appearing brain tissue. The metabolic profile of locations 1, 2 and 3 were not different and summarized for comparison with location 4

Probe location was assessed on all available head CT scans during CMD-monitoring by an independent neuroradiologist, blinded to clinical and neuromonitoring data, with respect to focal brain lesions (Fig. 1). *Perilesional* probe location was defined as the presence of a focal hyperdense (SAH-related parenchymal hemorrhage, thick SAH-clot) and/or focal hypodense (perioperative edema, perihematomal edema, other focal brain edema, initial ischemic stroke, periinterventional infarction, delayed cerebral edema) CT-lesion within 1 cm of the edge of the gold tip of the CMD-probe. Direct contact of the tip with a hypodense lesion was additionally subclassified as *intralesional* probe location, while CMD-data with probe location inside a hyperdense lesion were excluded from the analysis. If there was no focal lesion within 1 cm of the tip, probe location was graded as *normal*-*appearing* brain tissue. CMD-values of the 24 h preceding the CT scan were analyzed. For the assessment of temporal dynamics, we classified the CT scans into three groups (days 0–3, days 4–7 and after 7 days).

### Statistics

Continuous variables are reported as mean ± standard error of mean or median and interquartile range (IQR), as appropriate. Categorical variables are reported as count and proportions in each group. Time-series data were analyzed using generalized estimating equations (GEEs) with an autoregressive process of the first order to account for repeated measurements within subjects. The impact of probe location on cerebral metabolism was assessed in a linear GEE-model with the respective CMD parameter as dependent variable and dichotomized probe location as independent variable. In the same manner, the associations with functional outcome were analyzed by using the dichotomized outcome variable as independent variable. Statistical models were adjusted for important covariates as specified in the results. All analyses were performed with IBM-SPSS V24.0 (SPSS Inc., Chicago, IL, USA). Statistical significance was defined as *p* value < 0.05.

## Results

Baseline characteristics, hospital complications, and outcome of 51 SAH-patients are given in Table 1. Neuromonitoring was started on day 1 [1, 2] after SAH. CMD was performed for a median of 9 (IQR 6-13) days. In total, 143 head CT scans with a median of two scans [1–3] per patient were graded. Overall, 2259 CMD samples [median 39 (IQR 23-65) per patient] were analyzed.

**Table 1:** Study population

Characteristics, complications, outcome	Median (IQR) or *n* (%)
Age (years), median (IQR)	59 (50–67)
Gender (female), *n* (%)	36 (71)
APACHE II score, median (IQR)	16 (12–20)
Hunt and Hess grade (admission), *n* (%)	
2	4 (8)
3	12 (23)
4	6 (12)
5	29 (57)
Loss of consciousness (initial), *n* (%)	36 (71)
Modified Fisher grade, *n* (%)	
1	2 (4)
2	3 (6)
3	12 (23)
4	34 (67)
SAH-sum score, median (IQR)	23 (15–28)
IVH sum score, median (IQR)	4 (0–8)
Aneurysm size > 10 mm, *n* (%)	13 (26)
Global cerebral edema, *n* (%)	15 (29)
Hydrocephalus requiring EVD, *n* (%)	44 (86)
Clipping, *n* (%)	32 (63)
Pneumonia, *n* (%)	34 (67)
Sepsis, *n* (%)	21 (41)
Delayed cerebral ischemia, *n* (%)	16 (31)
Length of ICU stay (days), median (IQR)	33 (23–49)
Modified Rankin scale (after 3 months), *n* (%)	
0	2 (4)
1	9 (18)
2	3 (6)
3	7 (14)
4	8 (15.5)
5	14 (27)
6	8 (15.5)

*APACHE* acute physiology and chronic health evaluation, *EVD* external ventricular drain, *ICU* intensive care unit, *IQR* interquartile range, *IVH* intraventricular hemorrhage, *SAH* subarachnoid hemorrhage

CMD-probes were detected in *normal*-*appearing brain tissue* on 53/143 scans (37%) and in *perilesional* location on 90/143 scans (63%). There was no difference in metabolite concentrations between intralesional and perilesional probe location, except for a higher LPR (*p* = 0.04) in intralesional hypodense probe location in close vicinity (within 1 cm of the tip) to a hyperdense lesion.

**Fig. 2:**
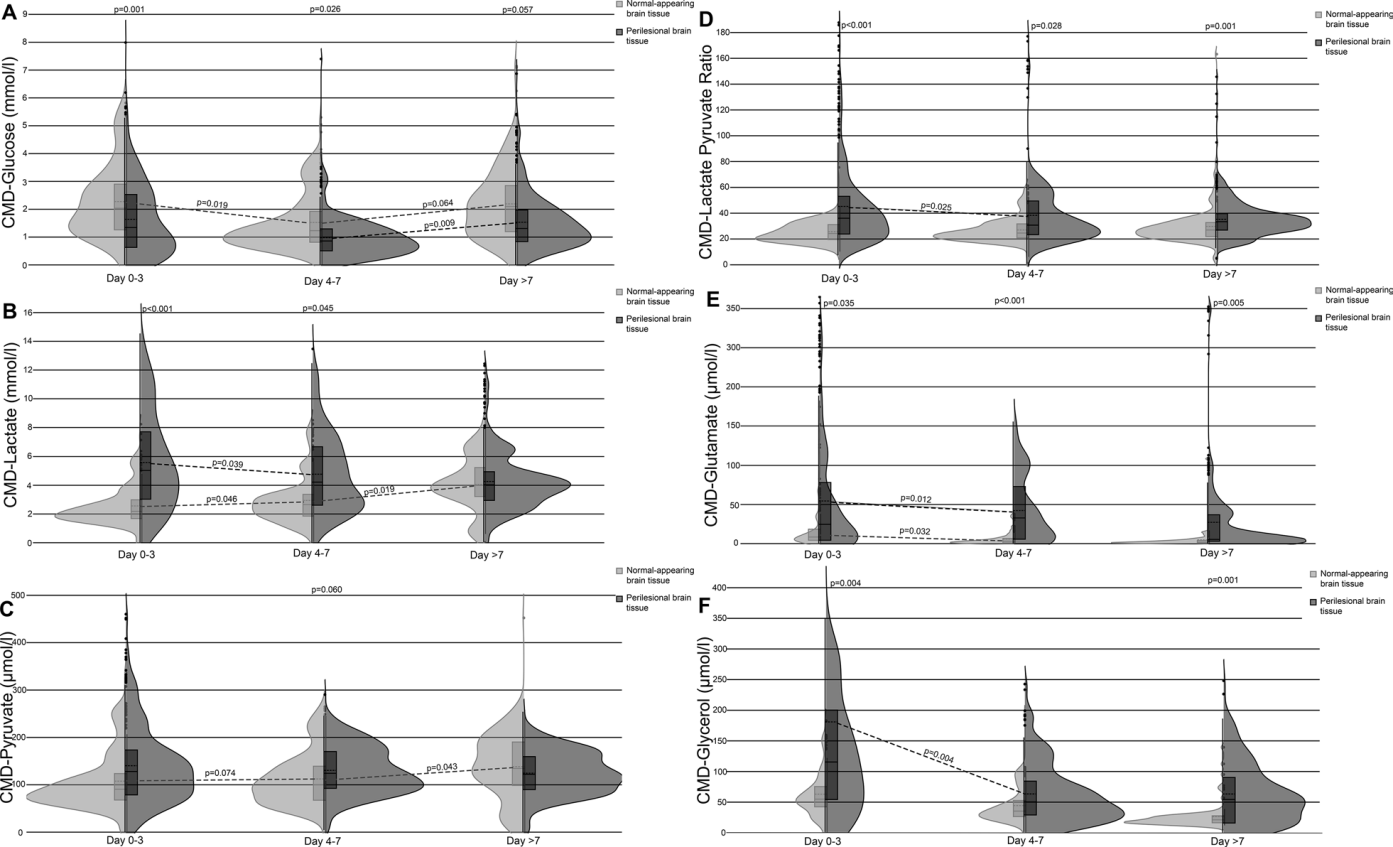
Violin plots depicting concentrations and temporal dynamics of CMD-glucose (**a**), CMD-lactate (**b**), CMD-pyruvate (**c**), CMD-LPR (**d**), CMD-glutamate (**e**), and CMD-glycerol (**f**) in normal-appearing brain tissue (light gray) and perilesional brain tissue (dark gray); the x-axis indicates time from SAH in three groups [days 0–3 (24 CT scans and 273 samples in normal-appearing tissue, 37 CT scans and 481 samples in perilesional tissue), days 4–7 (20 CT scans and 363 samples in normal-appearing tissue, 23 CT scans and 418 samples in perilesional tissue), and after day 7 (9 CT scans and 189 samples in normal-appearing tissue, 30 CT scans and 535 samples in perilesional tissue)]; the outer line of the plots shows the distribution of all data points. Boxes indicate median and IQR. The dashed line within the boxes represents the mean. *p* values above the plots describe the significance of differences between normal-appearing and perilesional brain tissue during the group of days specified below. Dashed lines connecting plots indicate significant changes over time (*p* values shown next to lines); statistical analyses were performed using a linear model in GEEs with the respective CMD parameters as dependent variable. Models were adjusted for age, gender, Hunt and Hess grade, and cerebral perfusion pressure. All *p*-values < 0.1 are reported. *CMD* cerebral microdialysis, *LPR* lactate-to-pyruvate ratio, *IQR* interquartile range

Cerebral perfusion pressure (CPP) did not differ between measurements in normal-appearing and perilesional brain tissue (*p* = 0.484, supplemental Figure). Mean values of CMD parameters in normal-appearing and perilesional brain tissue are shown in Table 2. CMD-glucose levels were lower in perilesional probe location (*p* = 0.003), whereas CMD-lactate (*p* < 0.002), CMD-LPR (*p* < 0.001), CMD-glutamate (*p* = 0.002), and CMD-glycerol levels (*p* < 0.001) were higher. Temporal dynamics of cerebral metabolism are shown in Fig. 2. Neuroglucopenia (27.1 vs. 8.8%, *p* = 0.002), metabolic distress (33.1 vs. 8.6%, *p* = 0.002), and mitochondrial dysfunction (50.3 vs. 21.4%, *p* = 0.005) were more common in perilesional compared to normal-appearing brain tissue. All analyses were adjusted for CPP.

**Table 2:** Overall differences between normal-appearing and perilesional brain tissue

CMD parameter	Perilesional brain tissue [median (IQR)] *N* = 1434 CMD samples	Normal-appearing brain tissue [median (IQR)] *N* = 825 CMD samples	*p* value
CMD-glucose (mmol/l)	1.15 (0.66–1.90)	1.54 (1.02–2.63)	0.003
CMD-lactate (mmol/l)	4.24 (2.85–6.46)	2.74 (1.84–3.84)	0.002
CMD-pyruvate (µmol/l)	124 (88.7–168)	104 (70.4–150)	0.34
CMD-LPR	33.8 (24.8–45.9)	25.0 (20.5–31.7)	< 0.001
CMD-glutamate (µmol/l)	14.4 (3.60–52.8)	3.75 (1.49–9.28)	0.002
CMD-glycerol (µmol/l)	63.8 (30.9–123)	37.3 (24.0–62.3)	< 0.001

Statistical analyses were performed using a linear model in GEEs with the respective CMD parameters as dependent variable. Models were adjusted for cerebral perfusion pressure

*CMD* cerebral microdialysis, *GEEs* generalized estimating equations, *IQR* interquartile range, *LPR* lactate-to-pyruvate ratio

### Changing Brain Pathology in Relation to Probe Location

In 13 patients, we found a transition of CMD-probe location from normal-appearing to perilesional brain tissue by either the expansion of an existing focal pathology, the occurrence of a new focal lesion, or the dislocation of the catheter tip. CMD-glucose levels significantly decreased (1.58 ± 0.06 to 1.24 ± 0.06 mmol/l, *p* = 0.039), whereas CMD-glutamate (8.39 ± 1.18 to 24.33 ± 2.43 µmol/l, *p* = 0.006) and CMD-glycerol levels (39.98 ± 3.28 to 60.26 ± 3.59 µmol/l, *p* = 0.004) increased with the change in probe location. CMD-lactate levels non-significantly increased (3.44 ± 0.06 to 3.95 ± 0.19, *p* = 0.60), and CMD-pyruvate levels non-significantly decreased (137.9 ± 4.26 to 117.6 ± 3.29 µmol/l, *p* = 0.16), resulting in a trend toward higher LPR (28.28 ± 0.56 to 34.01 ± 1.31, *p* = 0.08). The frequency of metabolic distress increased from 6.2 to 21.3% (*p* = 0.046), and the frequency of neuroglucopenia increased from 7.2 to 30.0% (*p* = 0.01). All statistical models were adjusted for age, gender, Hunt and Hess grade, and CPP.

### CMD-Probe Location and Outcome

**Fig. 3:**
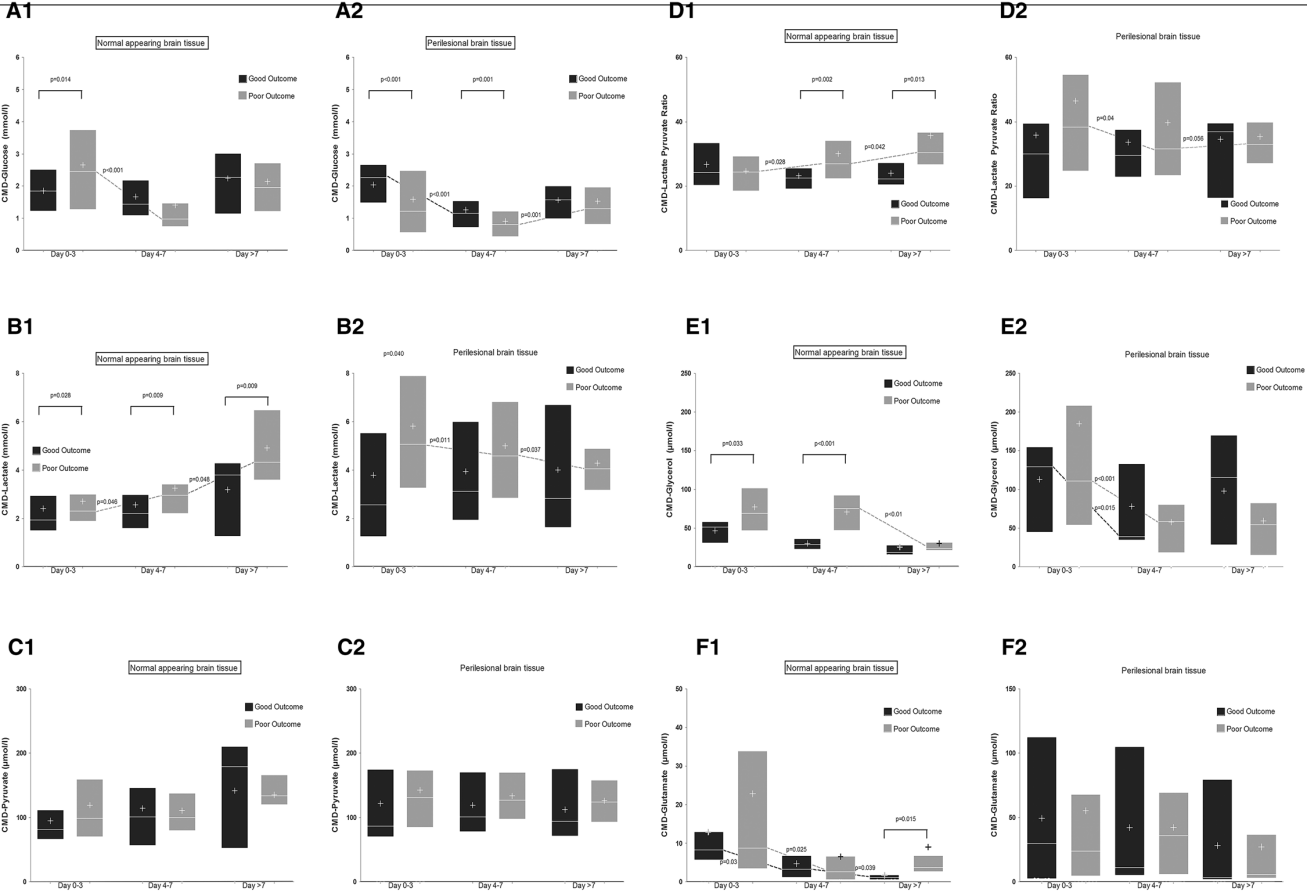
Median (IQR) and temporal dynamics of CMD-glucose (**a**), CMD-lactate (**b**), CMD-pyruvate (**c**), CMD-LPR (**d**), CMD-glycerol (**e**), and CMD-glutamate (**f**) in patients with good (black bars) and poor functional outcome (dark gray bars); plus signs within plots represent the mean; data are separately shown for normal-appearing brain tissue (1) and perilesional brain tissue (2). The x-axis indicates time from SAH in three groups: days 0–3 [184 samples of patients with good outcome (53 perilesional, 131 normal-appearing tissue) and 570 samples of patients with poor outcome (428 perilesional, 142 normal-appearing tissue)]; days 4–7 [246 samples of patients with good outcome (85 perilesional, 161 normal-appearing tissue) and 535 samples of patients with poor outcome (333 perilesional, 202 normal-appearing tissue)]; and after day 7 [149 samples of patients with good outcome (56 perilesional, 93 normal-appearing tissue) and 575 samples of patients with poor outcome (479 perilesional, 96 normal-appearing tissue)]; *p* values above the plots describe the significance of differences between patients with good and poor outcome during the group of days specified below. Dashed lines connecting plots indicate significant changes over time (*p* values shown next to lines); statistical analyses were performed using a linear model in GEEs with the respective CMD parameters as dependent variable. Models were adjusted for age, gender, Hunt and Hess grade, and cerebral perfusion pressure. All *p* values < 0.1 are reported. *CMD* cerebral microdialysis, *LPR* lactate-to-pyruvate ratio, *IQR* interquartile range

Patients with poor outcome after 3 months had lower CMD-glucose levels (1.54 ± 0.03 vs. 1.77 ± 0.04, *p* = 0.014) and higher levels of CMD-lactate (4.60 ± 0.06 vs. 3.08 ± 0.09, *p* = 0.001), CMD-LPR (37.5 ± 0.57 vs. 27.9 ± 0.65, *p* = 0.012), CMD-glutamate (33.7 ± 1.6 vs. 19.3 ± 1.7, *p* = 0.006), and CMD-glycerol (96.8 ± 5.3 vs. 48.5 ± 2.4, *p* = 0.004). There were no differences in CMD-pyruvate levels. Detailed information on temporal dynamics of CMD parameters with respect to probe location and functional outcome is provided in Fig. 3.

CMD-glucose levels were lower in patients with poor functional outcome in perilesional (*p* = 0.041), but not in normal-appearing brain tissue (*p* = 0.63). In perilesional brain tissue, these differences were more pronounced during the first week after SAH (*p* = 0.002). Interestingly, neuroglucopenia was significantly associated with poor functional outcome in either of the probe locations (*p* = 0.015 and *p* = 0.016).

CMD-lactate levels and CMD-LPR were higher in patients with poor functional outcome in normal-appearing (*p* = 0.014 and *p* < 0.001), but not in perilesional brain tissue (*p* = 0.35 and *p* = 0.56). Metabolic distress and mitochondrial dysfunction were strongly associated with poor outcome in normal-appearing (*p* = 0.001 and *p* = 0.002), but not in perilesional brain tissue (*p* = 0.51 and *p* = 0.69).

CMD-glutamate levels were higher in patients with poor functional outcome in normal-appearing (*p* < 0.001), but not in perilesional brain tissue (*p* = 0.57). CMD-glycerol levels were higher in patients with poor functional outcome in normal-appearing (*p* < 0.001), but not in perilesional brain tissue (*p* = 0.23).

All analyses were adjusted for age, gender, Hunt and Hess grade, and CPP.

### CMD and DCI

DCI was associated with lower CMD-glucose levels (*p* = 0.002) and neuroglucopenia (*p* = 0.004) in normal-appearing brain tissue, as well as a higher LPR (*p* = 0.001) and metabolic distress (*p* = 0.008) in perilesional brain tissue.

## Discussion

Our main findings are that the brain metabolic profile assessed by CMD largely depends on probe location, that lesion development can be detected by trend analysis of brain metabolites, and that probe location should be taken into account when CMD-derived parameters are used for neuroprognostication.

CMD is a local monitoring tool quantifying small metabolites in brain tissue surrounding the CMD-membrane (cylinder with a length of 1 cm) [21]. As shown in TBI-patients, perilesional tissue exhibited lower levels of CMD-glucose and higher levels of CMD-lactate, CMD-LPR, CMD-glutamate, and CMD-glycerol compared to normal-appearing brain tissue [6–8]. Therefore, probe location should be an integral part when interpreting brain metabolic changes assessed by CMD. So far, probe positioning has been neglected in most reports due to the lack of a precise definition based on conventional imaging techniques [2]. Here, we propose a simple method using clinical routine head CT scans and describe metabolic profiles in perilesional and normal-appearing brain tissue.

CMD-glucose levels were lower in perilesional location when compared to normal-appearing brain tissue. Absolute brain interstitial glucose levels are difficult to interpret as they depend on complex interactions including systemic glucose delivery, glucose transport across the blood–brain barrier and diffusion in brain tissue as well as cerebral energy consumption. We previously investigated the effect of enteral nutrition on brain glucose levels in SAH-patients and detected a significant increase independent of probe location [2]. However, absolute concentrations of CMD-glucose were significantly lower in perilesional brain tissue [2]. In the current study, we found a higher incidence of neuroglucopenia in the proximity of focal lesions. This may be explained by a decreased cerebral blood flow in hypodense areas (on head CT scans) [22], or glucose hypermetabolism, as previously reported in SAH-patients with GCE and in the perihematomal area of intracerebral hemorrhage patients [23, 24]. In the current study, we found neuroglucopenia to be associated with poor outcome irrespective of probe location; however, we found that absolute CMD-glucose levels measured in normal-appearing brain tissue may not accurately predict outcome. Based on the expert recommendation to treat pathologically low CMD-glucose levels, our findings are important for future prospective interventional trials [1]. However, the effect of a liberal glucose regimen may result in minor increases when CMD-glucose is measured in the perilesional area [2]. Moreover, trend analysis revealing decreasing CMD-glucose levels may indicate the development of new focal brain lesions as previously described [12].

Not only cumulative mean CMD-lactate and CMD-LPR levels, but also their temporal dynamics significantly differed between probe locations. The initial peak and consecutive decrease in CMD-lactate levels and CMD-LPR in perilesional tissue is reminiscent of the pattern found in SAH-patients with acute focal neurological deficits on admission [25]. Elevated CMD-lactate levels have traditionally been associated with anaerobic metabolism and poor outcome [2]. While our results confirm an association of overall higher CMD-lactate levels with poor outcome, integration of probe location confirmed this independent association primarily in normal-appearing brain tissue. Similar to CMD-lactate levels, elevated CMD-LPR is indicative for either cerebral ischemia or even more frequent, mitochondrial dysfunction [2, 26]. In our patients, metabolic distress and mitochondrial dysfunction were more common in perilesional brain tissue. As higher CMD-lactate levels and CMD-LPR were associated with poor outcome in normal-appearing brain tissue, an impaired oxidative brain metabolism seems to indicate adverse pathophysiologic processes beyond ischemia or focal lesions visible on head CT contributing to poor functional outcome. Recently, the metabolic profile of mitochondrial dysfunction was defined as elevated LPR together with normal-to-elevated pyruvate concentrations [26]. The impact of mitochondrial dysfunction on functional outcome is still unclear. We found it to be more common in the vicinity of focal lesions, but it was only associated with poor outcome in normal-appearing brain tissue. The incidence of highly elevated CMD-LPR, referred to as metabolic distress, however, significantly increased when new lesions developed, which is in line with the previous studies [12]. This may indicate that the frequency of the occurrence of metabolic distress is a more sensitive marker of emerging brain pathology than absolute LPR values, which did not significantly increase before new lesions were detected on CT scans. Elevated CMD-lactate levels do not only result from ischemia, but also from hyperglycolysis, which was described in SAH-patients with GCE [23]. In contrast to hypoxic/ischemic lactate, hyperglycolytic elevated lactate has even been associated with good functional outcome [27], possibly explaining the missing association between CMD-lactate levels and CMD-LPR in perilesional brain tissue. In summary, the reason for the lack of an association between metabolic derangement/mitochondrial dysfunction with functional outcome in perilesional brain tissue remains speculative. Metabolic distress in the perilesional area may simply represent the pathology associated with focal brain lesions and not (or less) reflect the metabolic condition of the whole brain. In contrary, if metabolic distress and/or mitochondrial dysfunction is detected in radiologically normal-appearing brain tissue, this may reflect global pathologies including generalized edema and generalized relative hypoperfusion [3], high-grade neuroinflammation [28], and axonal damage [29], which may better prognosticate functional outcome.

In the current consensus statement, the measurements of CMD-glutamate and CMD-glycerol were regarded as having questionable relevance for clinical patient management [1]. CMD-glutamate is a marker of excitotoxicity, with excess levels being associated with (and potentially contributing to) ischemia and poor outcome. CMD-glycerol is a marker of cellular decay. Here, we describe an association of elevated CMD-glutamate and CMD-glycerol levels with poor outcome in normal-appearing, but not in perilesional brain tissue. Similar to our observations regarding CMD-lactate and CMD-LPR, this indicates that monitoring brain metabolism may capture tissue damage not depicted by CT-imaging. Furthermore, there was a much larger elevation in CMD-glutamate and CMD-glycerol levels in the proximity of focal brain lesions. In line with this, higher CMD-glutamate and CMD-glycerol concentrations were previously described in patients with focal neurological deficits; however, the authors did not report a spatial relation of brain lesions and the CMD-probe [11, 25]. Our observation of a pronounced increase in CMD-glutamate and CMD-glycerol associated with the development of new focal lesions in the vicinity of the CMD-probe may strengthen the indication for measuring these parameters bedside, especially as we found only a non-significant trend toward higher CMD-LPR levels.

We are aware of limitations using CT scans for grading probe location, which are not sensitive enough to detect minor ischemic or hemorrhagic pathologies which would potentially be visible using MRI. Unsurprisingly, we found a slightly different metabolic profile with lower CMD-pyruvate levels and a higher CMD-LPR in *normal*-*appearing* brain tissue compared to the previously reported profile in healthy brain tissue of uninjured patients [30]. Further limitations include patient population, which was a highly selected cohort of poor-grade SAH-patients undergoing invasive neuromonitoring. Still, our data reflect clinical practice of CMD in poor-grade ventilated patients. The volume of tissue sampled by a CMD-catheter is a cylinder with a height of 10 mm and a diameter of a few millimeters [21]. With respect to these technical suppositions and guided by the previous studies, we used a distance of 1 cm from the CMD-probe to distinguish normal-appearing from perilesional tissue. Furthermore, we did not use all available CMD-data, but only the 24-h preceding CT scans, which may influence outcome analyses. For this reason, we also chose not to further investigate the associations between CMD parameters and DCI in different probe locations.

Our findings support the use of CMD as tool to detect brain metabolic changes associated with secondary brain injury. Further research is needed to identify potential treatment targets derived from metabolic monitoring. Treating neuroglucopenia appears to be a suitable approach, as it was associated with poor functional outcome irrespective of probe location and the development of new focal brain lesions. Our data indicate that CMD-probe placement in normal-appearing brain tissue may yield more relevant information than placement in perilesional tissue. The integration of brain metabolic parameters may further increase the sensitivity of neuroprognostication after acute brain injury.

## Conclusions

Focal brain lesions significantly impact on absolute concentrations and temporal dynamics of CMD parameters. The prognostic value of most CMD-derived metabolites seems to be limited to measurement in normal-appearing brain tissue. In contrast, neuroglucopenia remains a strong prognostic indicator independent of probe location. CMD was sensitive to detect the development of new focal lesions in vicinity to the probe. Based on our findings, probe location should be described in research reporting brain metabolic changes measured by CMD and integrated in statistical models.

## Electronic supplementary material

Below is the link to the electronic supplementary material.

Supplemental Figure 1 shows mean (± standard deviation) CPP corresponding to CMD parameters assessed in normal-appearing and perilesional brain tissue. There was no difference between groups (p=0.484). CPP = cerebral perfusion pressure; CMD = cerebral microdialysis; (TIFF 1564 kb)
