# Supplementary figures and images for: The Importance of Probe Location for the Interpretation of Cerebral Microdialysis Data in Subarachnoid Hemorrhage Patients
Source: Neurocrit Care. 2019 Apr 29;32(1):135–44. doi: 10.1007/s12028-019-00713-8 (PMC7012974; doi:10.1007/s12028-019-00713-8)

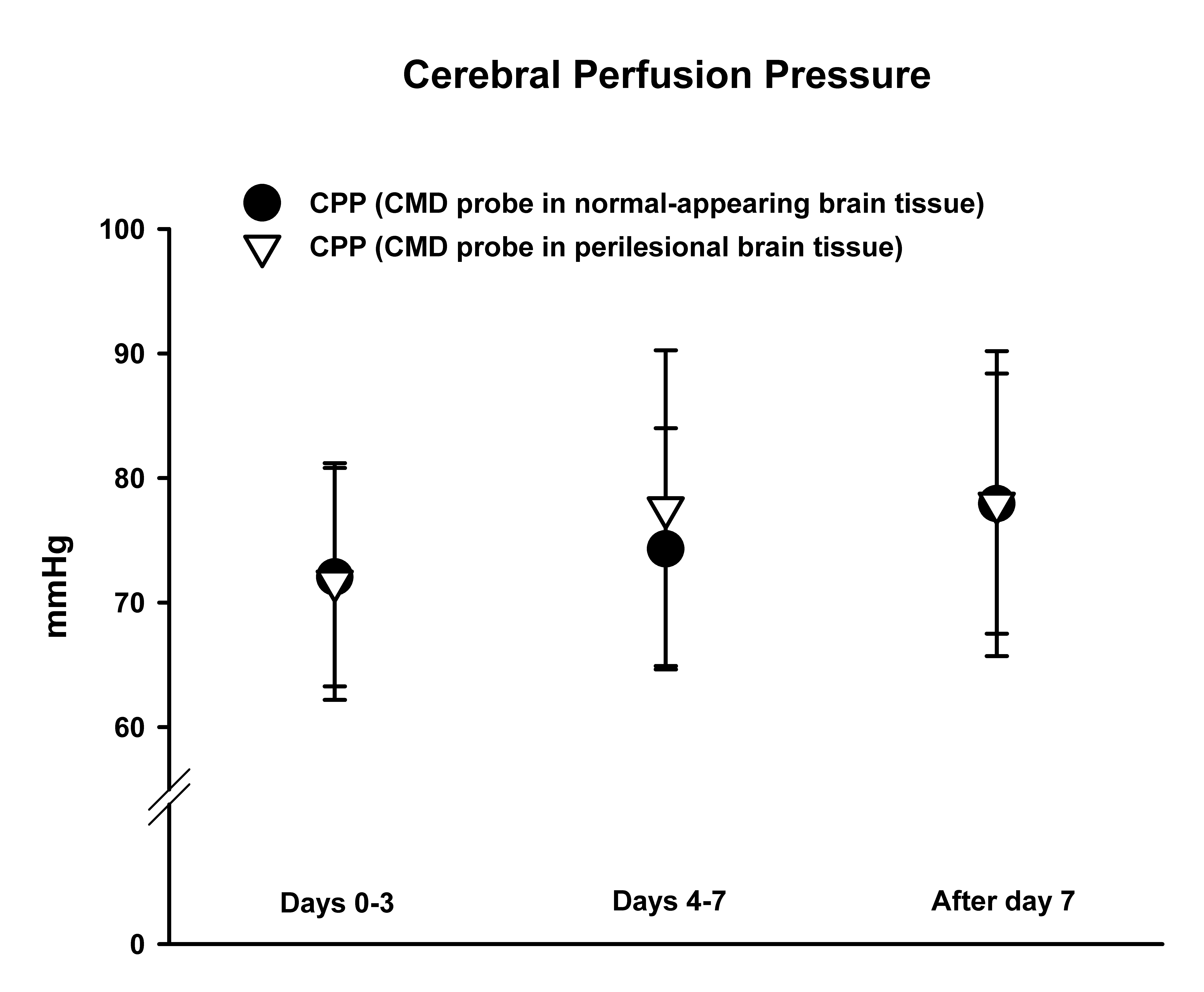

Supplement: Supplementary file 1 — Supplemental Figure 1 shows mean (± standard deviation) CPP corresponding to CMD parameters assessed in normal-appearing and perilesional brain tissue. There was no difference between groups (p=0.484). CPP = cerebral perfusion pressure; CMD = cerebral microdialysis; (TIFF 1564 kb) [file 12028_2019_713_MOESM1_ESM.tiff]
